# Hirayama disease: an uncommon cause of motor neuron disease

**DOI:** 10.1055/s-0046-1816039

**Published:** 2026-03-19

**Authors:** Trajano Aguiar Pires Gonçalves, Gustavo Novelino Simão, Rodrigo Siqueira Soares Frezatti, Fabio Silveira dos Santos Filho, Pedro José Tomaselli

**Affiliations:** 1 Universidade de São Paulo, Faculdade de Medicina de Ribeirão Preto, Departamento de Neurociências e Ciências do Comportamento, Ribeirão Preto SP, Brazil.; 2 Universidade de São Paulo, Faculdade de Medicina de Ribeirão Preto, Departamento de Imagens Médicas, Hematologia e Oncologia Clínica, Ribeirão Preto SP, Brazil.

**Keywords:** Neuromuscular Diseases, Motor Neuron Disease, Hirayama Disease

## Abstract

We herein report the case of a 23-year-old man with a 3-year history of progressive right-hand weakness, leading to functional impairment. A neurological examination revealed signs of lower cervical motor neuron involvement, including the reverse split hand sign and the Wartenberg's sign. Electrophysiological studies showed reduced compound muscle action potentials (CMAPs) in the right ulnar and median nerves, with preserved sensory conduction and neurogenic changes in the myotomes from C7 to T1. A conventional cervical spine magnetic resonance imaging (MRI) scan was unremarkable, but a flexion MRI scan revealed anterior displacement of the posterior dura and spinal cord compression, confirming the diagnosis of Hirayama disease (HD), which is a rare, self-limiting cervical myelopathy in young male patients caused by dynamic compression during neck flexion. The diagnosis requires a high index of suspicion and flexion MRI scans. While cervical stabilization remains controversial, it may help prevent progression in selected cases. The current report highlights the clinical and radiological features of HD, discusses differential diagnoses, and underscores the importance of dynamic imaging in young patients with asymmetric upper-limb weakness.

## CLINICAL VIGNETTE

**Figure 1:**
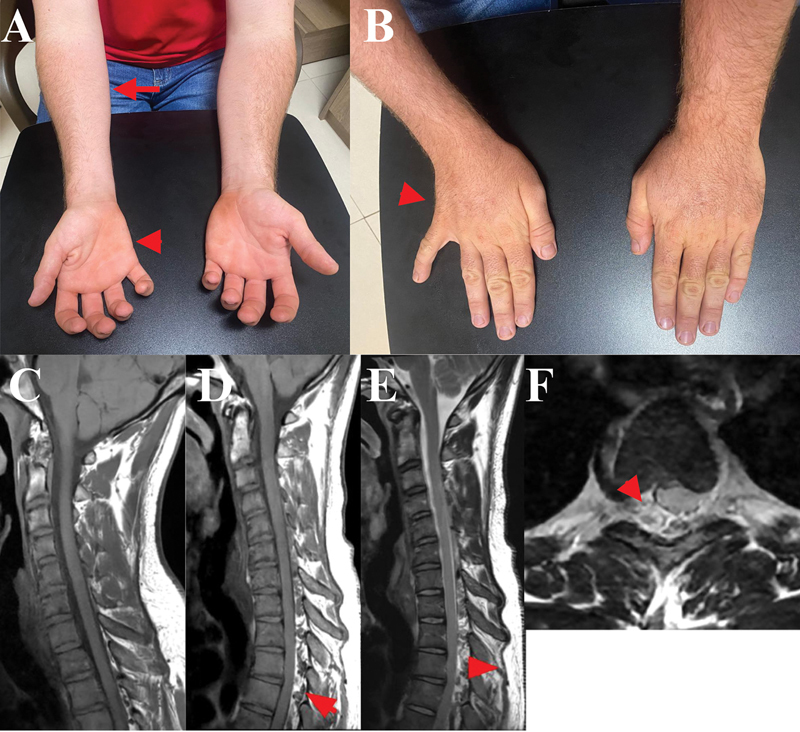
( **A** ) Atrophy of the right forearm flexor compartment (arrow) and right hypothenar atrophy (arrowhead). ( **B** ) Atrophy of the intrinsic muscles on the ulnar side of the right hand, which, together with hypothenar atrophy (arrow), characterizes the *reverse split hand sign* . Abduction of the fifth finger is also observed, consistent with Wartenberg's sign. Sagittal T1-weighted image (WI) in the neutral position ( **C** ), showing no significant abnormalities. Sagittal T1-WI in flexion ( **D** ), revealing anterior displacement of the dorsal dura (arrows), compressing the thecal sac, along with a crescent-shaped dorsal epidural compartment (arrows). Sagittal T2-WI ( **E** ) further demonstrating prominent flow voids and asymmetric spinal-cord compression.

A 23-year-old man presented with a 3-year history of painless, slowly-progressive weakness in the right hand, eventually leading to functional impairment. A neurological examination revealed atrophy of the intrinsic hand muscles, predominantly affecting the ulnar compartment (reverse split hand sign), marked weakness in fifth finger adduction, resulting in resting abduction (Wartenberg's sign), and atrophy of the forearm flexor compartment with sparing of the brachioradialis muscle ( Figure 1A,B ).

Nerve conduction studies demonstrated reduced CMAPs in the right median and ulnar nerves, with more prominent involvement of the ulnar nerve. The sensory conduction studies were normal. Needle electromyography showed reduced motor-unit recruitment with increased amplitude and duration of motor-unit potentials, without spontaneous activity, in the right forearm flexors, abductor pollicis brevis, and first dorsal interosseous muscles. Other muscles examined showed no abnormalities.

The conventional cervical spine magnetic resonance imaging (MRI) scan was unremarkable. However, dynamic MRI performed during neck flexion revealed anterior displacement of the posterior dura mater, consistent with dynamic spinal-cord compression, thus confirming the diagnosis of HD ( Figure 1C–E ).

The present study involved human participants and was approved by the ethics committee of Hospital das Clínicas da Faculdade de Medicina de Ribeirão Preto, Universidade de São Paulo (HCFMRP-USP). The participant provided informed consent to participate in the study.

## FROM PRESENTATION TO RESOLUTION: LESSONS LEARNED

### What is HD and what causes it?

Hirayama disease is a rare form of juvenile muscular atrophy predominantly affecting young males. It is attributed to dynamic cervical spinal-cord compression during neck flexion. 1 The prevailing hypothesis suggests that, during flexion, the posterior dura mater shifts anteriorly, compressing the cervical spinal cord against the posterior aspect of the vertebral body. This leads to dynamic compression of the anterior spinal artery, followed by chronic ischemia of the anterior horn cells—most notably at the levels from C7 to T1. 1 2

### What is the clinical presentation, and how does dynamic compression influence the phenotype?

The clinical prototype of HD is characterized by insidious-onset, asymmetric or bilateral weakness and atrophy affecting the upper limbs, primarily involving the flexor compartment of the forearm and the intrinsic hand muscles. The distribution typically follows the myotomes from C7 to T1 myotomes. 1

Motor neurons innervating the hypothenar region appear particularly susceptible to apoptosis, which explains the pronounced weakness and atrophy in the ulnar portion of the hand. 1 This feature underlies the so-called *reverse split hand sign* , a clinical clue that may help distinguish HD from amyotrophic lateral sclerosis (ALS). 1 As a result of this pattern, patients frequently exhibit impaired adduction of the fifth digit, leading to an abducted posture of the little finger, known as Wartenberg's sign ( Figure 1B ). Preservation of the brachioradialis muscle in this setting is also a distinguishing feature. Sensory symptoms are typically absent or nonspecific. 1

The identification of the reversal split hand sign holds clinical significance in the evaluation of suspected motor neuron disease. This finding is typically observed in patients with ulnar neuropathy. It is uncommon in patients with ALS, occurring in approximately 4% of the cases. 3 In contrast, the classic split hand sign, characterized by selective atrophy involving the thenar eminence (due to weakness of the abductor pollicis brevis, which is innervated by the median nerve) and the first dorsal interosseous muscle (which is innervated by the ulnar nerve), represents a pattern of muscle wasting that does not conform to the anatomical territories of individual peripheral nerves. 3 This sign is observed in up to 70% of ALS patients, and it is considered a hallmark of the disease. 3 However, its interpretation must always take into account the broader clinical context, as it has also been described in other motor neuron disorders, such as postpolio syndrome and spinal muscular atrophy, as well as in certain peripheral neuropathies, including X-linked Charcot-Marie-Tooth disease type 1 (CMTX1; *GJB1* mutation). 3 4 Recognition of the atrophy pattern characteristic of the reversal split hand sign, and its distinction from the split hand sign, in the setting of suspected motor neuron disease, is highly suggestive of HD.

### How is the diagnosis of HD confirmed, and what are the expected findings in complementary investigations?

The diagnosis is established through the correlation of clinical findings with imaging evidence of dynamic cervical spinal cord compression occurring during neck flexion. 1 While standard cervical MRI may reveal subtle abnormalities—such as straightening of cervical lordosis, lower cervical-cord atrophy, or detachment of the posterior dura from the subjacent lamina—these findings are often nonspecific and difficult to identify without cervical flexion imaging. 1 5 An MRI performed during neck flexion more clearly demonstrates the forward displacement of the posterior dural sac, cord flattening, and a crescent-shaped high-intensity signal consistent with engorged epidural venous plexuses. These findings typically reverse in the neutral position and are associated with lower cervical-cord compression ( Figure 1D–E ). 1 5

Electrophysiological studies can support the diagnosis and help differentiate HD from other disorders ( Table 1 ). Nerve conduction studies often reveal reduced CMAPs in the ulnar and median nerves, with a decreased ulnar/median CMAP ratio—opposite to what is typically seen in ALS. 6 7 Sensory nerve conduction remains normal in most cases. 6 Needle electromyography shows neurogenic recruitment patterns in the lower cervical myotomes. Resting activity may vary, but fibrillation potentials and positive sharp waves are more frequently observed during the active progressive phase of the disease. 1 6 7

**Table 1:** Differential Diagnosis of HD 1 12

	Hirayama disease	Cervical spondylotic myelopathy	Neurogenic thoracic outlet syndrome	Amyotrophic lateral sclerosis
Epidemiology	Young male subejcts aged 12 to 20 years	Middle-aged or elderly male subjects (40–60 years of age)	Young female subjects	Mean age of onset of symptoms in the sixth to seventh decades of life; Predominantly affects males
Clinical findings	Weakness in the forearm flexor compartment with preservation of the brachioradialis; Reverse split hand sign; Pyramidal signs are rare; Sensory function is preserved	Predominant involvement of proximal muscles, such as the deltoid and biceps;* Pyramidal signs may be present in all four limbs; Cervical pain is common	Predominant weakness and atrophy in the C8–T1 myotomes, most evident in the abductor pollicis brevis; Gilliatt-Sumner hand;** Hypoesthesia in the medial cutaneous territory of the forearm and the ulnar side of the hand; Cervical and shoulder pain are common; Palpation may reveal a cervical rib	Weakness with a myotomal pattern that may affect any body segment randomly; Split hand sign; Coexistence of pyramidal signs with muscle atrophy is characteristic; Sensory function is preserved
EMG	Reduced CMAP in the hands; Low ulnar/median ratio; Normal sensory conduction study	Reduced or normal CMAPs, with neurogenic recruitment on needle EMG; Normal sensory nerve conduction studies	Reduced CMAPs in the median and ulnar nerves, more pronounced in the median nerve when recorded from the abductor pollicis brevis; Abnormal sensory nerve conduction in the median, ulnar, and medial antebrachial cutaneous nerves, with preservation of the lateral antebrachial cutaneous nerve and more pronounced abnormalities in the ulnar and medial antebrachial cutaneous nerves	Reduced CMAPs beyond the upper limbs, with a high ulnar/median ratio; Normal sensory nerve conduction studies
Radiology	Static cervical MRI may show straightening of the cervical spine and subtle detachment of the posterior dura from the subjacent lamina; Cervical MRI during neck flexion may enhance the visualization of posterior dural detachment, flow voids, and dynamic compression of the lower cervical spinal cord	Spinal canal narrowing, which may be associated with T2/FLAIR signal changes at the site of compression; in more severe cases, contrast enhancement may be observed (pancake sign)	Neuroaxis MRI is usually normal; Plexus MRI may reveal predominant compression of the lower trunk of the brachial plexus	Spinal and cranial MRI findings are usually unremarkable. However, cranial MRI may reveal SWI hypointensity in the motor cortex—known as the “band sign”—as well as T2/FLAIR hyperintensity along the pyramidal tract.

Abbreviations: CMAP, compound muscle action potential; EMG, electromyography; FLAIR, fluid-attenuated inversion recovery; MRI, magnetic resonance imaging; SWI, susceptibility-weighted imaging.

Notes: *However, in the distal subtype, the intrinsic hand muscles may also be involved. **Gilliatt-Sumner hand is characterized by a constellation of symptoms that include predominant atrophy of the abductor pollicis brevis muscle, additional atrophy of the interosseous and abductor digiti minimi muscles, and preserved sensation in the area innervated by the median nerve (including the thenar eminence). This pattern is considered a diagnostic indicator of neurogenic thoracic outlet syndrome.

### What is the prognosis and management of HD?

Generally a self-limiting condition, in most cases, HD progresses over a period of 3 to 5 years, after which neurological deficits tend to stabilize. 1 Nevertheless, early diagnosis followed by appropriate intervention may improve functional outcomes. 1

The mainstay of treatment strategies is cervical stabilization. Continuous use of a cervical collar is initially recommended as the first-line approach. This strategy has been associated with subjective improvement in muscle strength, reduction in cold paresis (a transient worsening of weakness triggered by exposure to cold, frequently seen in HD but also reported in motor neuropathies such as multifocal motor neuropathy), and shortening of the progressive phase when compared with patients who did not use the collar, making it the most commonly-employed treatment for HD. 1 However, given the requirement for continuous use, 24 hours per day for 3 to 4 years, long-term adherence is often difficult. In an observational study, 8 adequate compliance was reported in approximately 57% of the patients (8 out of 14); all compliant patients achieved clinical stabilization, whereas 4 out of the 6 non-compliant patients experienced disease progression.

In recent years, growing evidence has supported surgical cervical stabilization as an alternative in selected cases. Surgical treatment has been shown 1 9 to provide objective improvement in muscle strength and to shorten the duration of disease progression. It is currently indicated for patients with progressive disease despite collar use, intolerance to prolonged collar wear, or severe symptoms at onset. Several surgical techniques have been described, including laminectomy, duraplasty, corpectomy, discectomy, decompression, and fusion. Meta-analyses 9 have reported higher success rates with anterior cervical fusion with plating, although no significant difference has been demonstrated between the anterior and posterior approaches overall.

These approaches are increasingly supported by evidence, with observational studies 10 indicating that cervical stabilization may prevent further deterioration and, in more than half of the patients, even lead to mild improvement in muscle strength. Adult patients with a history of typical HD and stable symptoms may be managed conservatively, although rare cases of recurrent weakness have been reported. 11

In conclusion, progressive monomelic atrophy and weakness in young patients may encompass a wide range of differential diagnoses, including radiculopathies, plexopathies, and even motor neuron disease. The identification of typical atrophy patterns, such as the reversal split hand sign, flexor forearm compartment atrophy, and preservation of the brachioradialis muscle, in the appropriate clinical context, should strongly suggest HD, which must then be confirmed through cervical spine MRI with neck flexion. Although it is a self-limiting condition, early diagnosis is crucial, as there is increasing evidence that cervical stabilization measures may contribute to disease stabilization and shortening of the progressive phase.

## Notes

TEACHING POINTS

1. Intrinsic hand muscle weakness and atrophy in the ulnar compartment, with forearm flexor atrophy and preserved brachioradialis, may indicate Hirayama disease (HD).
2. Subtle cervical magnetic resonance imaging (MRI) findings may occur, but a normal scan does not exclude the disease. Imaging must include neck flexion.
3. A reversed ulnar/median compound muscle action potential (CMAP) ratio, with the ulnar values reduced relative to the median value, is a key electrodiagnostic clue for HD disease, contrasting with the typical amyotrophic lateral sclerosis (ALS) pattern.
